# A behavioral study on tonal working memory in musicians and non-musicians

**DOI:** 10.1371/journal.pone.0201765

**Published:** 2018-08-02

**Authors:** Yue Ding, Kathleen Gray, Alexander Forrence, Xiaoqin Wang, Juan Huang

**Affiliations:** 1 Laboratory of Brain and Intelligence and Department of Biomedical Engineering, Tsinghua University, Beijing, P.R. China; 2 Peabody Institute, The Johns Hopkins University, Baltimore, MD, United States of America; 3 Department of Biomedical Engineering, The Johns Hopkins University, Baltimore, MD, United States of America; Universidad de Salamanca, SPAIN

## Abstract

Tonal working memory (WM) refers to the maintenance and the online manipulation of tonal information and has been suggested to involve different mechanisms than verbal WM. Previous research has suggested that verbal WM performance is determined by the duration instead of the number of verbal materials. We investigated in the present study to what degree that the number and the duration of notes in a sequence influence the tonal WM in participants with or without professional musical training. The forward tonal discrimination task in Experiment 1 tested the maintenance of the tonal information and the backward N-back tonal task in Experiment 2 probed the running memory span of tonal information. Results show that the number of notes, but not the duration of notes in a tone sequence significantly affects tonal WM performance for both musicians and non-musicians. In addition, within a minimum musical context, musicians outperformed non-musicians in the N-back tonal task but not the forward tone sequence discrimination task. These findings indicate that the capacity of tonal WM is determined by the number of notes but not the duration of notes in a sequence to be memorized, suggesting a different mechanism underlying tonal WM from verbal WM. Furthermore, the present study demonstrated that N-back tonal task is a quantitative and sensitive measure of the effect of musical training on tonal WM.

## Introduction

Working memory (WM) refers to a cognitive system where information is temporarily stored with a limited capacity that allows for online manipulation of the maintained information [1–3]. Auditory WM stores both phonological information that form verbal representations such as words and phonemes, and non-phonological information such as pitch, speech prosody, and timbre [1,4,5]. The latter is referred to as the tonal WM. Behavioral studies have suggested that the tonal WM involves different mechanisms than the verbal WM [4,6–8]. For example, the interference between two verbal WM tasks is stronger than the interference between a verbal WM task and a tonal WM task [7,8]. Another piece of supporting evidence is that amusics exhibit deficits in the pitch memory but not in the verbal memory [9–14]. Neural imaging studies have shown that certain brain areas are specifically involved in the tonal WM but not in the verbal WM [15–18].

An important question addressed by researchers studying auditory WM is whether the number of items or the duration of stimulus determines the capacity limit of WM [19–21]. It has been shown that participants’ performance in verbal WM tasks depends on the length of a verbal sequence to be memorized; the longer the sequence, the poorer the performance [3,19]. The effect of sequence length has also been examined in other studies, showing that participants’ memory span for sequences consisting long words was smaller than short words with fewer syllables or shorter duration, the so-called “the word length effect” [3,20,21]. However, regardless of the difference between verbal and tonal WM, whether the number of notes or the length of note sequence or both, determines the capacity of tonal WM remains unclear.

It has been shown that the capacity of verbal WM is modulated by the articulatory time of the words. A few studies have investigated the influence of tone duration on tonal WM. which was referred to as the “music length effect” in parallel to the “word length effect” in verbal WM. Akiva-Kabiri et al. (2016) examined the effect of music length on tonal WM by testing participants in a tonal discrimination task [22]. They found that both the length of tone sequences and the rate of presentation (tempo) influenced participants’ performance and a significant interaction between the two factors was also observed. Schulze (2012) showed that both musician and non-musician’s WM performance decreased as the length of tone sequence increased [23]. Albouy et al. (2016) reported that pitch memory in amusics increased with increased tone duration [24]. However, other studies have challenged these findings. Using six-tone sequences without explicit musical structure as the testing materials, Li et al. (2013) showed that only the number of notes defined the capacity of tonal WM [25]. In the present study, we re-examined this issue and investigated the factors that influence tonal WM performance by varying the number of notes systematically and changing the duration of notes in auditory tone sequences independently. We hypothesize that if the number of notes is the factor that determines the capacity of tonal WM, participants’ WM performance should not be significantly different between a quarter note sequence and an eighth note sequence of the number of notes but different length. On the other hand, if the length of a tone sequence is the factor that determines the capacity of tonal WM, participants’ WM performance should not be different between a quarter note sequence and an eighth note sequence of the same length but different number of notes.

When measuring the limit of verbal WM, two types of tasks have been used in previous studies, e.g., forward digit span or sentence recognition tasks for the capacity of the storage and maintenance of information [26,27], and backward digit span or word identification for the online manipulation of remembered verbal information [28,29]. The forward paradigm was commonly used to study tonal WM [22,25]. Dowling investigated tonal WM in non-musicians using forward standard melody, inversion of pitch contour of standard melody, and backward retrograde transformation of standard melody as testing materials [30]. Using forward and backward sequence recognition tasks, Schulze and colleagues studied the influence of musical structure on WM performance in musicians and non-musicians [23]. These studies, however, only tested the maintenance and manipulation of tone information in terms of pitch contour perception. The memory limit of individual tones was not directly revealed by responding if the orders of tone sequences were the same or different. While the N-back paradigms being a typical task for investigating verbal WM capacity [29,31,32], was rarely reported in the measurement of tonal WM [25]. The N-back paradigm evokes several WM processes such as monitoring, updating, and manipulation of remembered information and it has been commonly used as an assessment to measure WM capacity [29,31–35]. To examine the processes of individual tones, we adopted in the present study both forward tonal discrimination task and backward N-back tonal task in assessing the tonal WM performance.

Musical training is believed to be an influential factor on WM performance, especially for a music-related ability such as tonal WM. Studies have shown that the processing of structured and unstructured tone sequences is different in musicians, but not in non-musicians [15,36–38]. Lerdahl and Jackendoff reported that musical grouping rules help segment a sequence of tones into its constituent subgroups [39], which could significantly enhance musicians’ performance in certain tonal WM tasks. This notion was supported by findings by other researchers that musicians performed better in WM tasks using tonal rather than atonal sequences as testing materials [15,40–44], suggesting that musicians’ knowledge of musical regularities plays a role in tonal WM. We further examined these issues in the present study. To test the influence of music structure on WM performance, Experiment 1 used two types of testing materials, the conventional musical sequences that strictly obey the regularities of Western tonal music and random tone sequences. In Experiment 2, we designed different types of adjacent notes to the target note using an N-back paradigm to avoid the interference of explicit knowledge of music on the tonal WM performance.

In summary, in the present study, we aimed to answer the question whether the number of notes or the length of note sequence defines the capacity of tonal WM. We assessed the capacity of tonal WM with two tasks, the forward tonal discrimination task (Experiment 1) that reflects the ability of the maintenance of tonal information, and the backward N-back tonal task (Experiment 2) that reflects the limits of the manipulating of individual tone information in tonal WM. We also examined the role of possible notes grouping in the manipulation of tonal sequence independent of the influence of explicit musical structure.

## Materials and methods

All experimental procedures conformed to the Declaration of Helsinki. This study was approved by the Institutional Review Board of the Johns Hopkins University.

### Experiment 1: Tonal discrimination task

#### Participants

Sixteen musicians and sixteen non-musicians were recruited from the Johns Hopkins University community and tested in Experiment 1. Musicians had at least 5 years of formal musical training (mean = 11.83 years, SD = 5.62 years), and were musically active at the time of the testing. None of the participants in the musician group reported possessing absolute pitch. For the non-musician group, the participants did not have any formal musical training apart from basic compulsory music classes prior to college. All participants reported having normal hearing. Some participants received course credit and others received a nominal fee for participating in testing sessions (up to 1 hour each session). Informed consent was obtained from all participants.

#### Stimuli

The experiments were conducted in a double-walled sound-attenuating chamber (IAC-1020). Acoustic stimuli were delivered to the participant via circumaural sealed headphones (HDA 200, Sennheiser, Old Lyme, CT) through a TDT RZ6 system (Tucker-Davis Technologies, Alachua, FL). Stimuli were delivered at a comfortable sound level (65–75 dB) adjusted according to each participant’s report. Testing protocols were executed with a custom MATLAB (Mathworks, Natick, MA) program developed in our laboratory.

**Fig 1:**
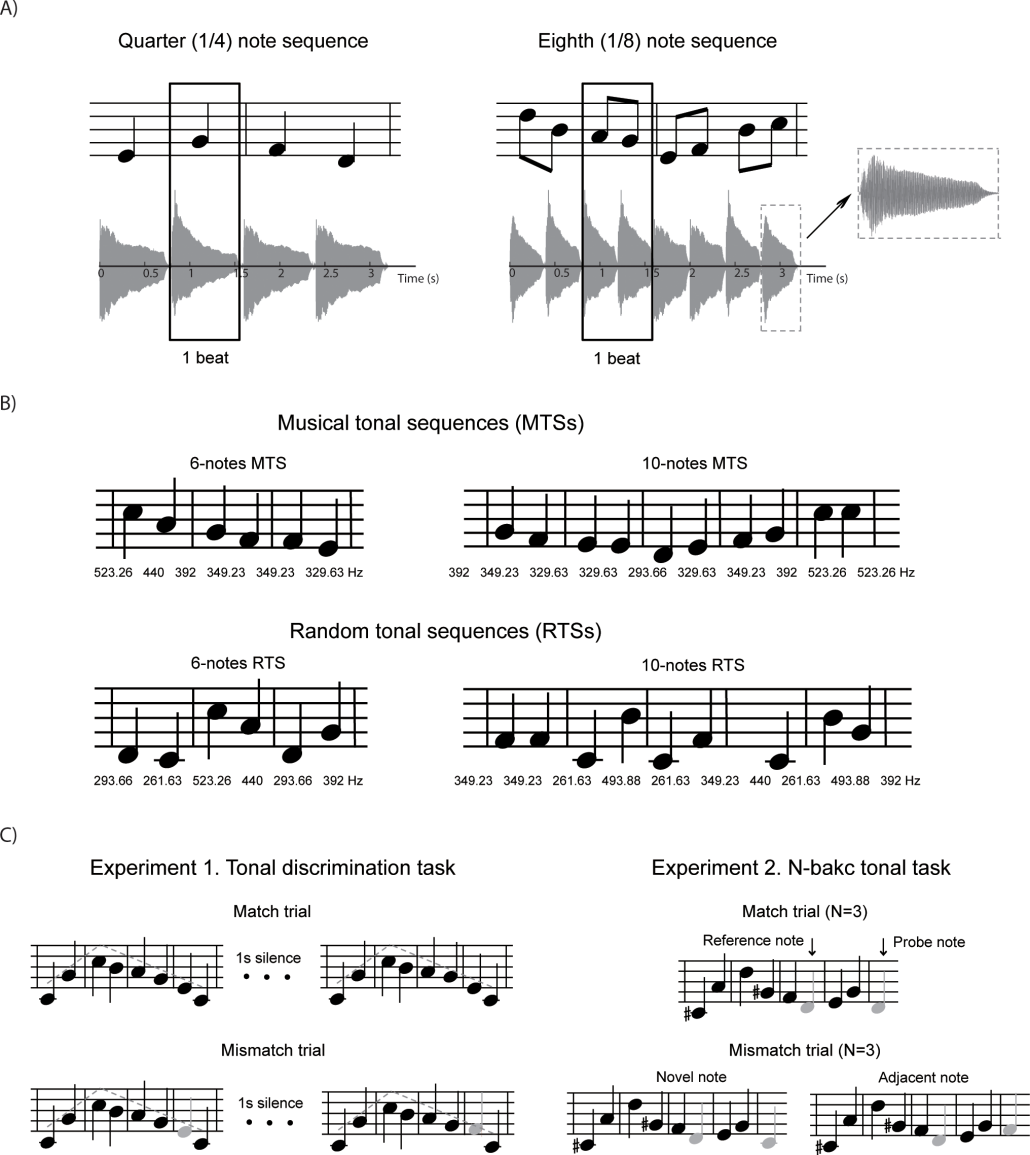
Examples of experimental stimuli and paradigm. (***A***) *Top plots*: Examples of a quarter (1/4) note sequence (*left*) and an eighth (1/8) note sequence (*right*) with two measures. *Bottom plots*: amplitude profiles of the note sequences shown on the top. Each measure consists of two beats, in which one beat corresponds to one note for the quarter note sequence and two notes for the eighth note sequence. (***B***) Examples of musical tone sequences (MTSs, *top plots*) and random tone sequences (RTSs, *bottom plots*) with 6 notes (*left*) or 10 notes (*right*). (***C***) Examples of testing sequence pairs. *Left*: Experiment 1, tonal discrimination task. *The upper row* is a match trial consisting of a pair of identical note sequences. *The lower row* is an example of mismatch trial, in which a note in the second sequence (gray note) is shifted up one semitone, but the pitch contour (dashed line) remains the same as the first sequence. The pitch shifts of mismatch trials happen in both directions, upwards or downwards. *Right*: Experiment 2, N-back tonal task. An example of N = 3 testing condition. *The upper row* is a match trial, in which the probe note (the last note) is the same as the reference note (the 3rd to the last note). *The lower row* are mismatch novel note trials where the probe note is different from any prior notes in the sequence. In the mismatch adjacent note trial (*right*) the probe note is the same as a note prior to the reference note ((N+1)th note condition).

Two types of tone sequences were used as testing materials: musical tone sequences (MTSs) and random tone sequences (RTSs). MTSs are tone sequences composed of seven musical notes from C major scale (C4: 261.63Hz, D4: 293.66Hz, E4: 329.63Hz, F4: 349.23Hz, G4: 392Hz, A4: 440Hz, B4: 493.88Hz) by a musician that are tonally structured as defined by the Western tonal system. RTSs are the repetitions of the same rhythm as those of MTSs, but with the notes randomly chosen from the same set of musical notes using a random permutation algorithm in MATLAB, where the tones do not have an obvious tonal structure (S2 Fig). Two musicians who did not participate in the experiments were asked to subjectively rate the tonality of the MRSs and RTSs using a 10 points scale in which 1 represents the least tonality and 10 represents the most tonality. Results show that RTSs were rated significantly lower than MTSs (two sample t-test, p<0.001). Fig 1B shows some MTSs and RTSs examples generated with the method described.

Each note was a synthesized pure tone (sine wave) modulated by an envelope that linearly increased from zero to the maximum amplitude at 1/8 of the note duration, and then linearly decreased to zero for the remainder of the note (Fig 1A, right inset). The modulation envelope of the notes produced a more natural timbre than that of pure tones. A testing sequence consisted of either 1/4 or 1/8 notes without gaps between notes (see Fig 1A). The note duration was 800 ms for 1/4 note and 400ms for 1/8 note. Testing sequences were presented to participants at the speed of 75 beats per minute (bpm), resulting in a beat duration of 800ms. Each bar (or measure) was a segment of 1600ms, corresponding to two beats or two notes for 1/4 notes and four notes for 1/8 notes. Each testing sequence contained between 1–6 measures, corresponding to 2–12 notes for the 1/4 note condition and 4–24 notes for the 1/8 note condition, respectively. Thus, the durations of sequences were the same for 1/4 and 1/8 note conditions. The testing sequences were randomly presented to participants. The two note conditions were tested in different blocks.

There were four testing blocks for each participant: 1/4 note MTS, 1/4 note RTS, 1/8 note MTS, and 1/8 note RTS. For each testing block, there were 6 measure conditions with 10 trials in each condition. 60 trials were completed for each testing block, and a total of 240 trials were completed for each participant in Experiment 1. In half of the trials, the pair of sequences was identical (“same trials”). For the other half of the trials, the pair of sequences was different only by one note, which was shifted up or down by one semitone (“different trials”). Only notes C, E, F and B from the seven original notes were selected for the pitch shift so that the “different trials” remained as musically structured for MTSs and randomly organized for RTSs. The overall pitch contours for the pair of sequences were the same in both types of trials (Fig 1C, left).

#### Procedures

During a trial, participants were asked to report whether the pair of sequences was the same or different by pressing one of two buttons on a response box. No feedback was provided to the participants. Participants’ responses and response times were recorded. For each trial, the two sequences were presented with a 1-second interval between them. The first sequence was the reference and the second sequence was the target. Participants could respond at any time when the target sequence was played. If a participant responded after the presence of the manipulated note in the “different trials” or after the last note in the “same trials”, the response was recorded as a valid response. If a participant responded before the presence of the manipulated note in the “different trials” or before the last note in the “same trials”, the response was recorded as an invalid response. Participants could decide how many blocks they would like to participate and when to terminate the testing. Data acquired from participants who completed all four testing blocks and with the valid response rate no less than 80% were included in the analysis reported here, which means each individual participant completed all 1/4 and 1/8 MTS and RTS testing blocks, and participant had at least 8 valid trials out of 10 trials for each bar/measure testing condition. Data from 13 musicians and 15 non-musicians in MTS tasks and 11 musicians and 11 non-musicians in RTS tasks were included in statistical analyses. The participant’s correct response rate was linearized to "rational arcsine units" (RAUs) by the rationalized arcsine transform [45].

### Experiment 2: N-back tonal task

#### Participants

Participant recruitment procedures and criteria were similar to those described in Experiment 1. Twenty-one musician and eighteen non-musician participants participated in Experiment 2. Some participants received course credit and others received a nominal fee for participation. Two sessions were tested. Each testing session consisted of 5 blocks and lasted up to 1 hour. Informed consent was obtained from all participants.

#### Stimuli

Twelve musical notes within one octave of middle frequency (ranging from 261.63 Hz to 493.88 Hz) were used to generate random tone sequences (e.g., C4, C^#^4/D^b^4, D4, D^#^4/E^b^4, E4, F4, F^#^4/G^b^4, G4, G^#^4/A^b^4, A4, A^#^4/B^b^4, and B4). Each sequence consisted of 9 to 12 notes with two note duration conditions (1/4 note and 1/8 note) which were randomly permutated except for the last note, which was manipulated based on the N conditions. The testing sequences generated from two note conditions and different numbers of notes were tested on each participant in a randomized order. The experiment was conducted in the same sound-attenuating chamber using the same experimental system as used in Experiment 1. Twenty-two participants tested in Exp 1 also participated in Exp 2, including 10 musicians and 11 non-musicians.

#### Procedure

During the experiment, participants were instructed to listen to the whole sequence carefully and then to identify whether the last note (the probe note) was the same or different from the reference note presented N notes prior to the last note in the sequence, referred to hereafter as the N^th^ reference note (Fig 1C, right). Participants indicated their response by pressing the “same” or “different” button on a response box. N was set to 2, 3, 4, 5, or 6 for each testing block based on results from pilot experiments to avoid floor and ceiling effects. For each session, half of the trials contained a probe note being identical to the N^th^ reference note. These trials were referred to as “match trials”. The other half of the trials were “mismatch trials”, among which 50% of trials had the probe note being a novel note (i.e., different from any notes presented prior to it), 25% trials had the probe note being the same as the (N + 1)^th^ note and 25% trials had the probe note being the same as the (N + 2)^th^ note (Fig 1C, right). We referred to these three types of “mismatch” trials as “novel note” and “adjacent note” ((N + 1)^th^ or (N + 2)^th^ note) conditions (Fig 1C, right). There were no more than two identical notes in any of the sequences. The experiment consisted a total of 10 testing blocks (N = 2, 3, 4, 5, or 6), 5 each for 1/4 note and 1/8 note conditions. Testing sequences in each block had a fixed N but a different number of notes (9, 10, 11, and 12) with 10 randomly generated copies and presented to participants in a randomized order. Before each testing block, participants were told the number of N of that block, so that they knew where the reference note they were looking for to compare with the last note. A total of 40 testing sequences in a block were randomly generated for each note number condition (9, 10, 11, and 12 notes) with 10 repeats. Data were acquired from 16 musicians and 16 non-musicians. Participants that completed all testing blocks for either the 1/4 or 1/8 note condition or both, were included in further analysis. For the control analysis of training effect and mismatch trials, the corrected accuracy was calculated.

## Results

### Experiment 1: Tonal discrimination task

Using the forward discrimination paradigm (Fig 1C, left), we measured participants’ ability to maintain tonal information in their WM system, as well as the effect of the duration of sequence and the number of note on the WM performance. Participants were asked to discriminate a pair of tone sequences and report whether the sequences were the same or different. Two types of tone sequences were used as testing materials, musical tone sequences (MTSs) and random tone sequences (RTSs) (Fig 1B). With this experimental design, the influence of musical context and previous musical training on WM capacity was also examined.

We conducted an overall ANOVA on the participants’ response to examine the effects of measure number (the duration of sequence), note duration (quarter vs. eighth note), sequence type (MTS vs. RTS), and musical training (musician vs. non-musician) using the statistical tools of MATLAB. Results showed significant main effects of measure number (F_(5, 552)_ = 96.89, *p* < 0.01, $\eta_{p}^{2}$ = 0.467), note duration (F_(1, 552)_ = 71.08, *p* < 0.01, $\eta_{p}^{2}$ = 0.114), and musical training (F_(1, 552)_ = 55.92, *p* < 0.01, $\eta_{p}^{2}$ = 0.092) but not sequence type (F_(1, 552)_ = 0.31, *p* = 0.576, $\eta_{p}^{2}$ = 0.001). However, the interaction between musical training and sequence type was significant (F_(1, 552)_ = 13.32, *p* < 0.01, $\eta_{p}^{2}$ = 0.024, S1 Fig). In the MTS condition, musicians had significantly better performance than non-musicians (t_(334)_ = 6.461, p < 0.001), while in the RTS condition, there was no significant difference between the two groups (t_(262)_ = 1.656, p = 0.099). We thus examined the influence of the duration of note and the number of notes on WM performance on the two groups for MTS and RTS conditions separately.

#### MTS condition

**Fig 2:**
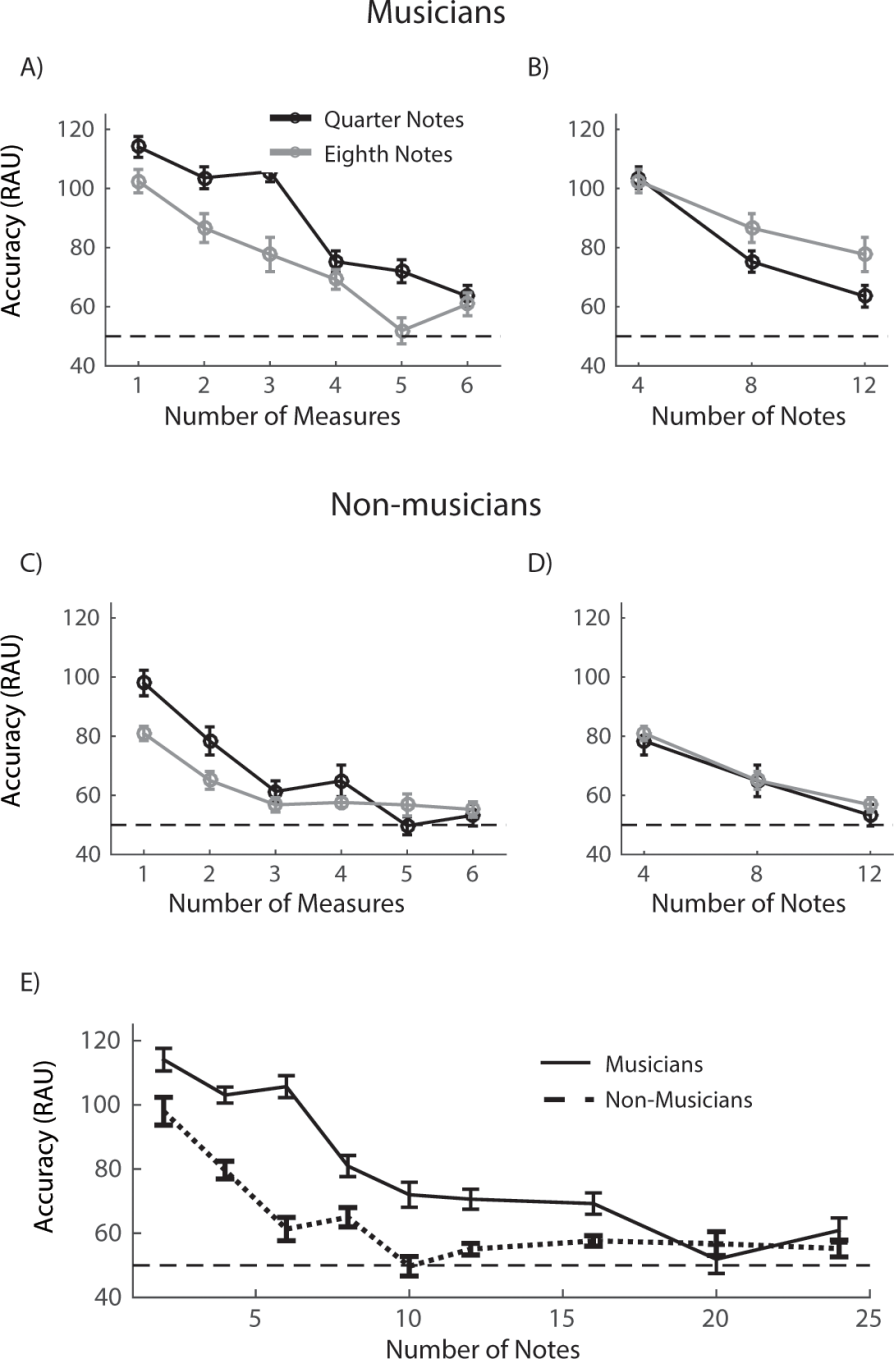
Results of tonal discrimination task in musical tone sequence condition. (***A***, ***C***) Performance of musicians (***A***) and non-musicians (***C***) as a function of the number of measures for quarter note (black curve) and eighth note (grey curve) conditions. The performance is plotted as the d-prime value. (***B***, ***D***) Performance of musicians (***B***) and non-musicians (***D***) as a function of the number of notes for quarter note (black curve) and eighth note (grey curve) conditions. (***E)*** Combined analyses of the quarter and eighth note conditions. Performance of musicians (solid curve) and non-musicians (dashed curve) as a function of the number of notes in a sequence. Horizontal dashed lines in all plots represent the chance level. Error bars are corrected S.E.M across participants. Stars mark the significant difference comparing to the chance level.

Fig 2 shows the results in the MTS condition. Fig 2A and 2C plot the performance of musicians (2A) and non-musicians (2C) as a function of the number of measures in a sequence. Repeated-measures ANOVA revealed significant main effects of music training (F_(1, 26)_ = 19.02, *p* < 0.01, $\eta_{p}^{2}$ = 0.422), the number of measures (F_(5, 130)_ = 56.05, *p* < 0.01, $\eta_{p}^{2}$ = 0.683) and note duration (F_(1, 26)_ = 27.52, *p* < 0.01, $\eta_{p}^{2}$ = 0.514). As the number of measures increases, participants’ performance declined in both groups. For a certain measure number, the quarter note condition contains only half of notes as in the eighth note condition, participants’ performance for quarter notes was better than eighth notes. For musicians (Fig 2A), the effects of measure number (F_(5, 60)_ = 33.54, *p* < 0.01, $\eta_{p}^{2}$ = 0.736) and note duration (F_(1, 12)_ = 23.92, *p* < 0.01, $\eta_{p}^{2}$ = 0.666) were both significant. Musicians’ RAU accuracy for quarter note sequences was better than that for eighth note sequences with two (t_(12)_ = 3.81, *p* < 0.05) and three measures (t_(12)_ = 3.59, *p* < 0.05, One-tail paired t-test with Bonferroni correction). For non-musicians (Fig 2C), the effect of measure number was significant (F_(5, 70)_ = 25.10, *p* < 0.01, $\eta_{p}^{2}$ = 0.642), as well as note duration (F_(1, 14)_ = 5.14, *p* < 0.05, $\eta_{p}^{2}$ = 0.269). Non-musicians had significantly higher RAU accuracy for 1/4 than 1/8 note sequences with only one measure (t_(14)_ = 3.32, *p* < 0.05).

We then compared participants’ performance between quarter and eighth notes conditions as a function of the number of notes in a sequence. As expected, RAU value dropped as the number of notes increased. Repeated-measures ANOVA showed that the number of notes significantly influenced the performance (F_(2, 52)_ = 41.54, *p* < 0.01, $\eta_{p}^{2}$ = 0.606). However, the participants’ performance for quarter and eighth note sequences was not significantly different (F_(1, 26)_ = 4.09, *p* = 0.054, $\eta_{p}^{2}$ = 0.121), for neither musicians (Fig 2B) nor non-musicians (Fig 2D).

We, therefore, combined the data of quarter and eighth conditions as a function of the number of notes in further analysis (Fig 2E). Repeated-measures ANOVA (between-participants factors: musicians vs. non-musicians; within-participants factors: 6 number of measures and 2 note durations) revealed a significant difference between musicians and non-musicians (F_(1, 26)_ = 15.43, *p* < 0.01, $\eta_{p}^{2}$ = 0.962). The musicians outperformed the non-musicians for note numbers up to 16 (*p* < 0.05, one-tail t-test with Bonferroni correction), except for note numbers of 2 and 8 (p > 0.05). Musicians’ RAU accuracy was significantly higher than the chance level at consecutive note numbers up to 16. Non-musicians’ performance drops faster than the musician participants and approached to the chance level when the note number was above 8. The results in Fig 2E show that both musicians and non-musicians are able to keep a certain number of musically organized notes in their WM to perform the tone sequence discrimination task, and that musicians could remember more notes at a time than non-musicians.

#### RTS condition

**Fig 3:**
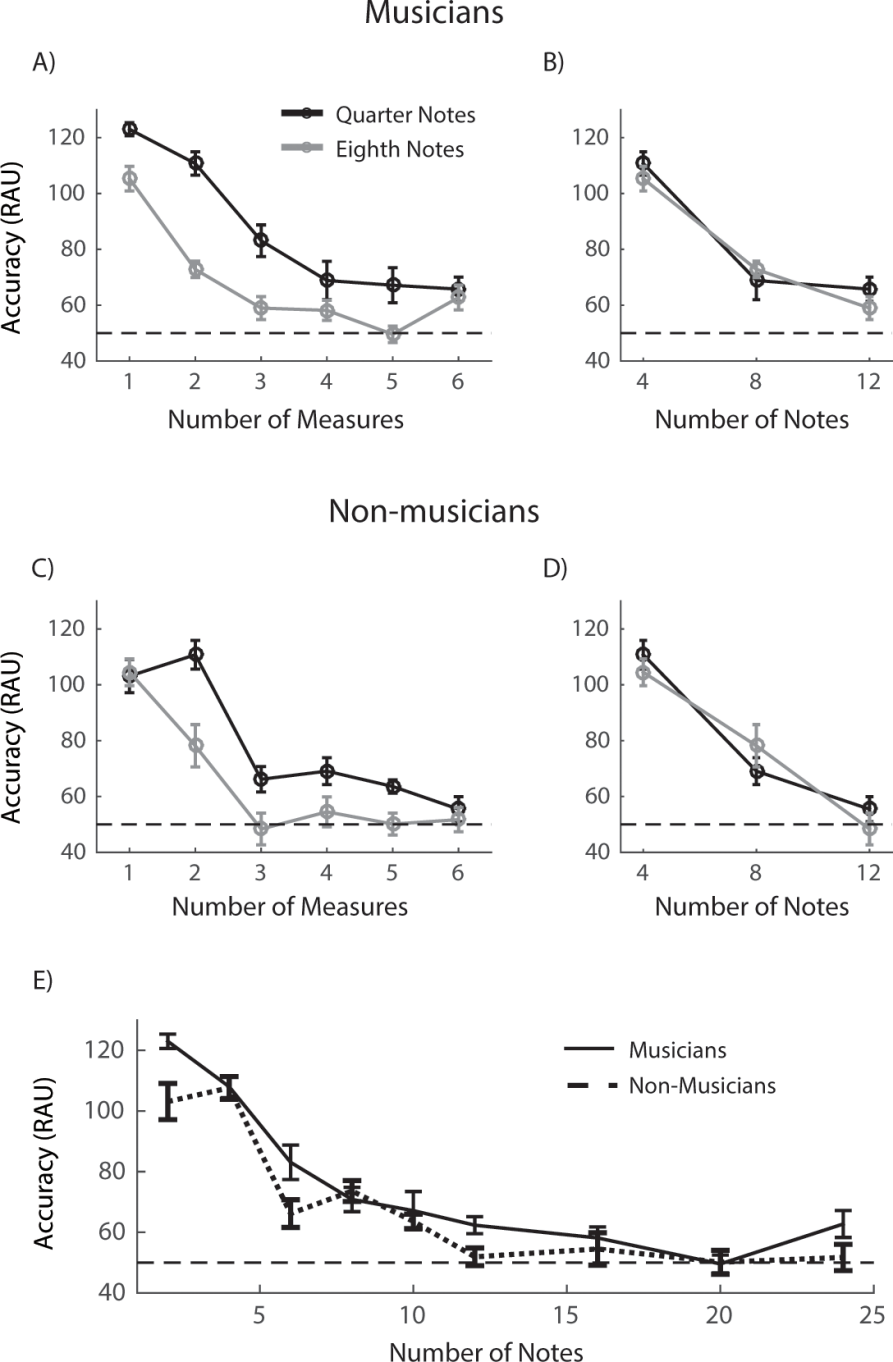
Results of tonal discrimination task in random tone sequence condition. (***A***, ***C***) Performance of musicians (***A***) and non-musicians (***C***) as a function of the number of measures for quarter note (black curve) and eighth note (grey curve) conditions. The performance is plotted as the d-prime value. (***B***, ***D***) Performance of musicians (***B***) and non-musicians (***D***) as a function of the number of notes for quarter note (black curve) and eighth note (grey curve) conditions. (***E***) Combined analyses of the quarter and eighth note conditions. Performance of musicians (solid curve) and non-musicians (dashed curve) as a function of the number of notes in a sequence. Horizontal dashed lines in all plots represent the chance level. Error bars are corrected S.E.M across participants. Stars mark the significant difference comparing to the chance level.

The testing sequences used in the RTS condition had no explicit musical structures (see Methods). Again, the participants’ performance declined as the number of measures and the number of notes increased for musicians (Fig 3A and 3B) and non-musicians (Fig 3C and 3D). However, no significant effect of note duration (quater vs. eighth note) was observed for both groups at a given number of notes. Repeated-measure ANOVA showed that both the number of measures (F_(5, 100)_ = 64.04, *p* < 0.01, $\eta_{p}^{2}$ = 0.762) and the note duration (F_(1, 20)_ = 38.38, *p* < 0.01, $\eta_{p}^{2}$ = 0.657) significantly influenced the participants’ performance. The performance for quarter note sequences was significantly better than eighth note sequences when the number of measures were 2 (t_(10)_ = 7.32, *p* < 0.05) and 3 (t_(10)_ = 3.46, *p* < 0.05) for musicians, and 2 (t_(10)_ = 3.81, *p* < 0.05) for non-musicians (one-tail paired t-test with Bonferroni correction). However, unlike the result of the MTS condition, musicians and non-musicians showed no significant difference in their WM performance with RTS sequences.

No significant effect of note duration (1/4 or 1/8) was observed for either musicians or non-musicians at a given number of notes. Thus, Fig 3E plots the results after combining the data of quarter and eighth conditions as a function of number of notes. Surprisingly, there was no significant difference between the two groups across all note numbers (Fig 3E, *p* > 0.05, two-tail two-sample t-test with Bonferroni correction), though the RAU accuracy was significantly above the chance level up to 16 notes for musicians and 10 notes for non-musicians (*p* < 0.05, one-tail t-test with Bonferroni correction). We conclude from the results of the RTS condition that when there was no explicit musical structure in a tonal sequence, musicians and non-musicians showed no significant difference in their WM performance, in contrast to the observation in the MTS condition (Fig 2E).

### Experiment 2: N-back tonal task

**Fig 4:**
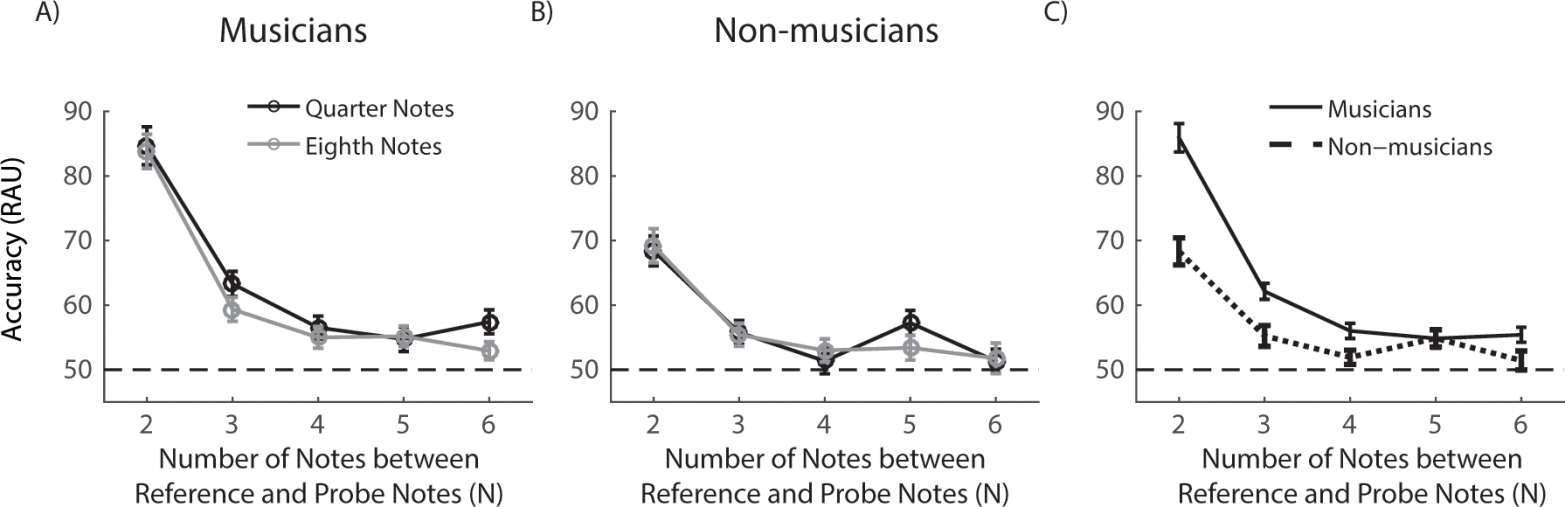
Results of N-back tonal task. (***A***, ***B***) Performance of musicians (***A***) and non-musicians (***B***) as a function of the notes between the reference and probe notes (N) for quarter note (black curve) and eighth note (grey curve) conditions. (***C***) Combined analyses of the quarter and eighth note conditions. Horizontal dashed lines in all plots represent the chance level. Error bars are corrected S.E.M across participants. Stars mark the significant difference comparing to the chance level.

In Experiment 2, we examined participants’ ability to recognize a reference note in a random tone sequence that was presented prior to a probe note using a backward N-back testing paradigm (Fig 1C, right), with the assumption that participants need to keep the ongoing tonal sequence in WM system in order to judge if the reference note matched the probe note. The number of notes presented between the reference note and the probe note is taken as the limit of WM capacity that allows for participants online manipulating or updating tonal information in their WM system. A univariate ANOVA was conducted that revealed a significant difference in performance between musicians and non-musicians (F_(1, 79)_ = 34.47, p < 0.001, $\eta_{p}^{2}$ = 0.028). When the data of the two groups were analyzed separately, the main effect of N was significant, for both musicians (F_(4, 316)_ = 50.94, *p* < 0.01, Welch F test) and non-musicians (F_(4, 39)_ = 23.87, *p* < 0.01, $\eta_{p}^{2}$ = 0.133). No difference between the quarter and eighth note conditions was observed for either group. Fig 4A (musicians) and 4B (non-musicians) plot RAU values as a function of the number of notes between the probe and reference notes. In Fig 4C, we combined RAU values calculated from results with the quarter and eighth note conditions. The musicians’ performance was significantly better than the chance level for N up to 6. Whereas the non-musicians’ performance was above chance level when N was 2, 3 and 5. Detailed comparison between the two groups across N conditions and the comparison between participants’ performance with chance level are show in Table 1.

**Table 1:** t-test analysis for Experiment 2.

N	Musician vs. Non-musician		Musicians vs. chance level		Non-musicians vs. chance level	
	t_(37)_	p	t_(17)_	p	t_(20)_	p
**2**	3.725	0.001	10.757	0.000	5.678	0.000
**3**	2.292	0.014	5.653	0.000	2.362	0.015
**4**	1.613	0.057	3.274	0.002	1.348	0.098
**5**	0.066	0.474	3.165	0.003	3.155	0.003
**6**	2.214	0.016	3.596	0.001	1.554	0.069

#### Error analysis

**Fig 5:**
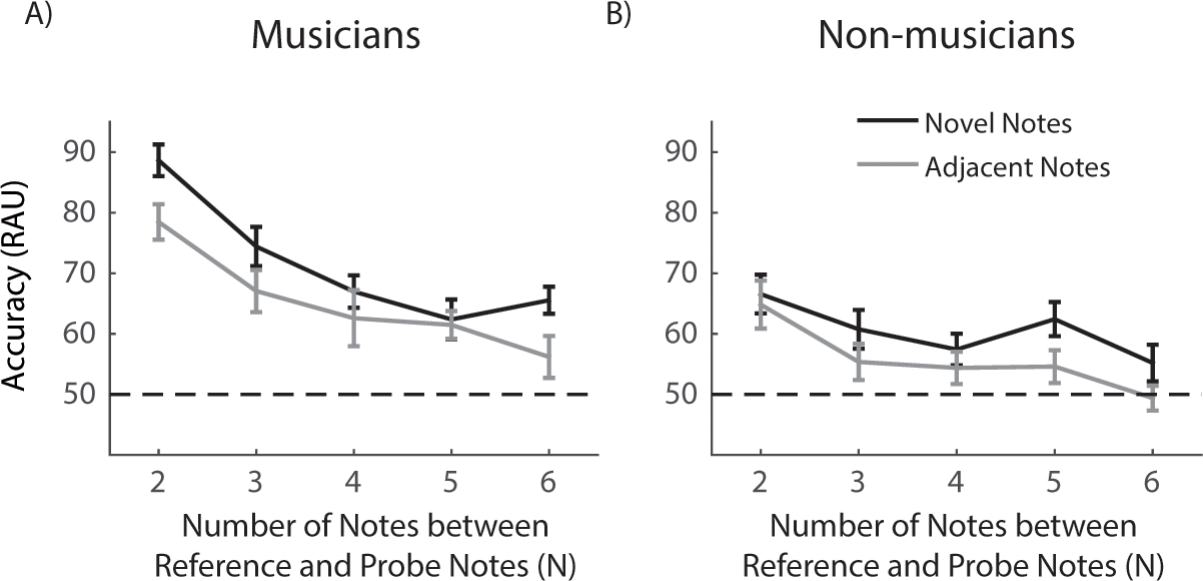
Comparison between the novel and adjacent notes in the N-back tonal task. Performance of musicians (***A***) and non-musicians (***B***) as a function of the notes between the reference and probe notes (N) when the probe note was a novel note (black curve) or identical to a note adjacent to the reference note (grey curve). The performance is plotted as the accuracy (RAU). Horizontal dash lines in all plots represent the chance level. Error bars are corrected S.E.M across participants.

We further analyzed the participants’ error patterns made with the two types of mismatch probe notes, novel and adjacent notes (see Methods, Fig 1C, right), in terms of RAU accuracy using the univariate ANOVA (N × probe note type). As shown in Fig 5A, for musicians, the RAU values in novel notes trials were significantly better than that in adjacent notes trials (F_(1, 9)_ = 4.48, *p* < 0.05, $\eta_{p}^{2}$ = 0.022). However, for non-musicians (Fig 5B), there was no significant difference between novel notes and adjacent notes trials (F_(1, 9)_ = 3.35, *p* = 0.069, $\eta_{p}^{2}$ = 0.020).

We concluded from the N-back tonal task results that musicians and non-musicians could only manipulate at least 5 and 2 notes respectively in their working memory system for further cognitive processing, regardless of the note duration, in consisting with our findings from Experiment 1. In addition, compared to non-musicians, musicians WM performance are more influenced by notes that adjacent to reference notes. These results indicate that the notes occurred at the adjacent position to a reference note interfered with the recognition of the reference note for musicians but not non-musicians, suggesting that musicians memorize tonal information in chunks whereas non-musicians memorize tonal information in individual notes.

#### Task-related learning effects

**Fig 6:**
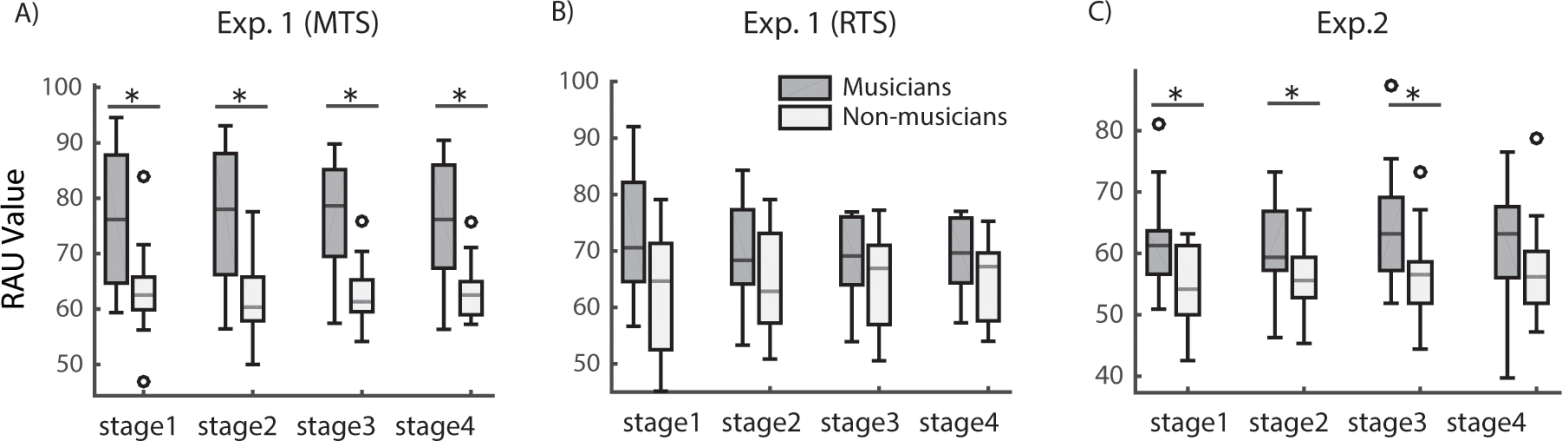
Performance across testing stages. Performance of musicians (gray box) and non-musicians (open box) is plotted for each of the four testing stages. The performance is plotted as the RAU value. Each box represents percentiles of the data. The line within the box indicates the median. Error bars represent the minimums and maximum values. Small circles indicate the outliers. The significant difference between musician and non-musicians groups is indicated by the asteroids above the two boxes for each testing stage. (***A***) MTS condition in Experiment 1. Musicians’ performance is significantly higher than non-musicians at all four testing stages. (***B***) RTS condition in Experiment 1. No significant difference between musicians and non-musicians. (***C***) Experiment 2. Musicians’ performance is significantly higher than non-musicians at stages 1, 2, and 3, but not at stage 4. The significant difference was marked as stars.

We have also evaluated the effects of task-related learning effects on WM ability by separating the trials into 4 stages based on the time of testing (Fig 6). There was no significant difference among the 4 testing stages in MTS (F_(1, 37)_ = 0.27, *p* = 0.69, $\eta_{p}^{2}$ = 0.01) and RTS tasks (F_(2, 39)_ = 0.17, *p* = 0.84, $\eta_{p}^{2}$ = 0.008) with Greenhouse-Geisser correction. Neither musicians nor non-musicians showed significant learning effect. Participants’ WM ability remained consistent across all 4 stages. More importantly, musicians’ out performed non-musicians at all 4 testing stages (Fig 6A) (stage 1: t_(26)_ = 3.27, *p* < 0.001; stage 2: t_(26)_ = 3.88, *p* < 0.001; stage 3: t_(26)_ = 4.76, *p* < 0.001; stage 4: t_(26)_ = 4.20, *p* < 0.001) only in the MTS condition (F_(1, 26)_ = 17.63, *p* < 0.001, $\eta_{p}^{2}$ = 0.404). No difference was found in any of the 4 stages in the RTS condition (Fig 6B) (stage 1: t_(26)_ = 1.76, *p* = 0.09; stage 2: t_(26)_ = 1.33, *p* = 0.20; stage 3: t_(26)_ = 1.31, *p* = 0.21; stage 4: t_(26)_ = 1.49, *p* = 0.15) between musicians and non-musicians (F_(1, 20)_ = 2.45, *p* = 0.13, $\eta_{p}^{2}$ = 0.109).

Analyses of Experiment 2 data are shown in Fig 6C. The effect of task-related learning or testing time order did not significantly influence subjects’ performance, F(3, 111) = 1.55, p = 0.21, $\eta_{p}^{2}$ = 0.04. No difference was observed at any of the 4 testing stages for either musicians or non-musicians. Again, the main effect of musical training was statistically significant (F(1, 37) = 10.15, p < 0.01, $\eta_{p}^{2}$ = 0.215). Musicians’ performance was significantly better than non-musicians (stage 1: t(37) = 2.98, p < 0.001; stage 2: t(37) = 2.21, p < 0.01; stage 3: t(37) = 2.90, p < 0.01), but not in stage 4 (t(20) = 1.79, p = 0.08).

These results further confirmed our conclusion on the music training effect we drawn from the analysis with RAU value comparison between the two groups, indicating that musicians’ WM outperformed non-musicians in both maintenance and manipulation of tonal information in tonal WM.

## Discussion

Previous studies have shown that the number of items and the articulation times of the items are two factors affecting verbal WM [19,21,46]. Researchers have reported that the articulation time significantly influences the capacity limits of verbal WM, indicating the major contribution of sub-vocal rehearsal to verbal WM [21,22]. In the present study, we systematically manipulated the number of notes in a sequence and the length of a tone sequence. As shown in Fig 2A and 2C (musical sequence) and Fig 3A and 3C (random sequence) for Experiment 1, participants’ sensitivity to tonal information decreased as the number of measures varied from 1 to 6 such that the length of tested sequences varied correspondingly between 1.6 and 9.6 seconds. The results seem to agree with the previous finding of “the word length effect” on the verbal WM capacity [20,21]. However, when the number of notes was fixed at 4, 8, or 12 (Fig 2B and 2D and Fig 3B and 2D), although the length of quarter note sequence was two times longer than that of eighth note sequence, participants’ WM performance was not different. In the N-back tonal task (Fig 4A and 4B), again, participants’ WM performance was found not different between the two note duration conditions. The present study does not reveal the sequence length effect. Neither the duration of note (quarter note vs. eighth note) in a tonal sequence nor the length of the note sequence significantly influenced participants’ performance when the number of note was fixed. The two duration conditions were chosen in our study (400 and 800ms) based on previous studies on the acoustically dominant time scale of a large dataset of music which shown that the peak of the temporal modulations frequency range of musical beats is at 0.5–3 Hz [47]. In addition, typical dancing music usually has the tempi of 94–176 beats per minute that corresponds to 1.6–2.9 Hz in envelope modulation [48]. The two durations chosen in current study correspond to different frequencies, e.g. 2.4 Hz and 1.25 Hz, represent a common situation in music, however, covers a small frequency range. That would limit the generalization of the conclusion to music with much slower or faster rhythmic pattern which could be much more difficult to memorize and thus possibly be influenced by the duration of notes. Nevertheless, the lack of difference in two very different durations we tested shed interesting light on how music might be processed by the brain.

Baddeley and colleagues observed that when the number of words in a sequence was fixed, the length of words was negatively correlated with participants’ performance [21]. The average articulation time difference in the study was between 150ms and 480ms with the average at 310ms. The duration difference between the quarter and eighth notes in the current study is 400ms, which is greater than the articulation time difference that has been shown to significantly influence the WM capacity for verbal words, however caused no significant difference on the WM performance. One possibility might be that the memory of tonal sequences relies less on sub-vocal rehearsal of individual notes than that of enunciable verbal syllables, but more influenced by the organization of notes that follow certain music grammar to form a contour of pitch variation. Therefore, the tonal WM is less influenced by the duration of notes but more determined by the number of notes.

The memory capacity limit is not a directly measurable quantity and can only be inferred by psychophysical experiments. The tonal discrimination task in Experiment 1 tests the immediate memory span by exposing a participant to a sequence of tonal notes. Participants need to maintain the tone information of the first sequence in order to judge if one of the tones in the second sequence is different. The N-back tonal task in Experiment 2, on the other hand, probes the running memory span by requiring participants to memorize a subset of the notes in a tone sequence in reverse order. Here we quantitatively measured the capacity of maintenance of tonal information. As measured in Experiment 1, the maximum number of notes in a sequence with the accuracy being above chance level is 16 for musical sequences (Fig 2E) and 8 (Fig 3E) for random sequences in musicians, and 8 for both musical and random sequences in non-musicians. Tested in Experiment 2, the maximum number of notes that musicians could effectively online process is 5, and non-musicians could only manipulate 2 notes at a time. The present study provides quantitative evidence supporting the notion that the number but not the duration of note or sequence, defines the capacity limit of tonal WM, no matter how much cognitive load involves in the process and independent of musical training experience. The current findings are consistent with prior work showing that the number of items defines the capacity limit of tonal WM [25] and vision WM [49]. Our results extend the understanding of the difference between verbal and tonal WM, provide a piece of supporting evidence of two independent mechanisms underlying verbal and tonal WM.

Since improving the ability of tonal information processing is one of the important goals of musical training, it’s reasonable to expect the musical training would significantly increase the capacity of tonal WM. In addition, the structure of note sequences would also be one of candidate factors that crucially influences tonal WM performance. Not surprising, the present study revealed the significant differences between musicians and non-musicians regarding the maximum number of notes that could be maintained and manipulated at a time. Using different categories of non-verbal sounds as the testing materials, Li et al. showed that the maximal number of sounds whose orders could be correctly recognized was slightly above three [25], in line with the range of our finding, i.e. 5 tones for musicians and 2 tones for non-musicians. Our findings are generally consistent with the previous finding by Halpern and Bower [40], which showed that musicians remembered musical sequences better than non-musicians. Some studies have suggested that musicians showed better performance in simple but not complex verbal WM tasks, say forward vs. backward verbal digit span task [50–52]. In the current work, we found that for tonal WM, musicians’ superiority existed regardless of task difficulty level. The enhanced tonal WM exhibited by the musicians likely resulted from long-term musical training. Although such long-term musical training produced enhancement could also be caused by better strategies of musical memory in musicians, such as integrating more notes or different features into single memory units as suggested by findings in visual WM literature [49]. Interestingly, however, musicians did not show better performance with the random tone sequences comparing with non-musicians (Fig 3E). One of the reasons contribute to this result might be the contour complexity difference between the MTs and RTs in which the former has relatively simpler contour whereas the latter has random contours. Musical training produced better musical skills exhibit more often in musical context where the contour of pitch variation remains relatively simple and agrees with musical grammar. Such a superior performance would less likely exhibit with note sequences that has random contour that do not form clear tonal structures.

It is reasonable to consider that one strategy to memorize and recall a tone sequence is by grouping a few notes together. In the tonal discrimination task (Experiment 1), the participants need to maintain the notes and the temporal order of the presented notes in their WM system in order to correctly perform the task. Although the musicians did not outperform non-musicians in the RTS condition (Fig 3E), their performance was significantly better in the N-back task. When adjacent notes were used as the probe notes, the musicians’ performance was significantly influenced, but not non-musicians’ performance (Fig 5A). It may due to the musical training that gave the musicians a better ability to group the notes that even not explicitly follow musical conventional structures. The results agree with Oberauer’s argument of binding processes being involved in order to successfully perform the N-back tonal task, suggesting that the influence of musical training on tonal WM not only exists in the musical context [53]. Our study suggests that the N-back tonal task might be a better method for evaluating the ability of tonal information processing in working memory.

The N-back task has been used to evaluate some other cognitive processes such as the conflicting processes between familiarity and recollection [53,54]. For example, if a current stimulus matches a previous stimulus but not the one N items previously presented in the sequence, the ability of resolving the conflict is required to perform the task, including inhibition and interference resolution [55]. Interestingly, our results show that the conflict resolving process only influenced musicians’ but not non-musicians’ WM performance. One explanation is that non-musicians’ performance was reaching a ground poor that could not be harmed further by the conflict solving processes. Another reason could be that only musicians established and maintained bindings among notes contained in their WM system that would affect the manipulation of tonal information. The enhanced tonal WM that we observed in musicians could also be caused by more efficient perceptual learning by musicians when exposed to the testing stimuli or practicing the tasks, considering tonal perceptual learning has previously been shown to be quite rapid [56]. However, the result of the analysis of the task-related learning effect indicates that the superior performance in musicians over non-musicians was not caused by short-term learning from doing the tasks (Fig 6).

The main finding of the present study is that the number of notes but not the duration of notes, determined the capacity of the maintenance and the manipulation of tonal information measured with both forward and backward tasks in both musicians and non-musicians. Tonal working memory is an important component of the auditory working memory that differs from verbal working memory. However, its underlying mechanisms remain unclear. It would be intriguing to compare the neural bases of verbal and tonal WM using the same experimental paradigm, such as the backward N-back task in the future. A recent imaging study by Kumar et al. (2016) demonstrated the involvement of the hippocampus in encoding, maintenance, and retrieval of tones in working memory, and the importance of the activity in the auditory cortex and inferior frontal gyrus for the maintenance of tone in working memory [57]. Kumar et al. (2016) study suggested that the long-range connectivity of the three brain areas may be responsible for keeping the representations of tone information active during working memory maintenance [57]. Future imaging and behavioral studies may identify brain areas that are specifically involved in tonal or verbal working memory processes.

## Supporting information

S1 Fig Subjective rating of musical tonality by two musicians. Y-axis: Average rating score. Scores are between 1–10, with 1 being least musical and 10 being most musical. Error bars are standard deviations. Dark bars: musical tonal sequences (MTSs). Gray bars: random tone sequences (RTSs). * p<0.001 (two sample t-test).
(TIF)

S2 Fig Trend analysis of the performance accuracy (RAU) between musicians and non-musicians with musical tone sequences (MTSs) and random tone sequences (RTSs). Musicians’ accuracy RAU value with MTSs was significantly higher (p<0.01) than that of non-musicians, whereas no significant difference was observed for test with RTSs. Error bars are standard errors.
(TIF)
