# Supplementary figures and images for: A behavioral study on tonal working memory in musicians and non-musicians
Source: PLoS One. 2018 Aug 2;13(8):e0201765. doi: 10.1371/journal.pone.0201765 (PMC6084024; doi:10.1371/journal.pone.0201765)

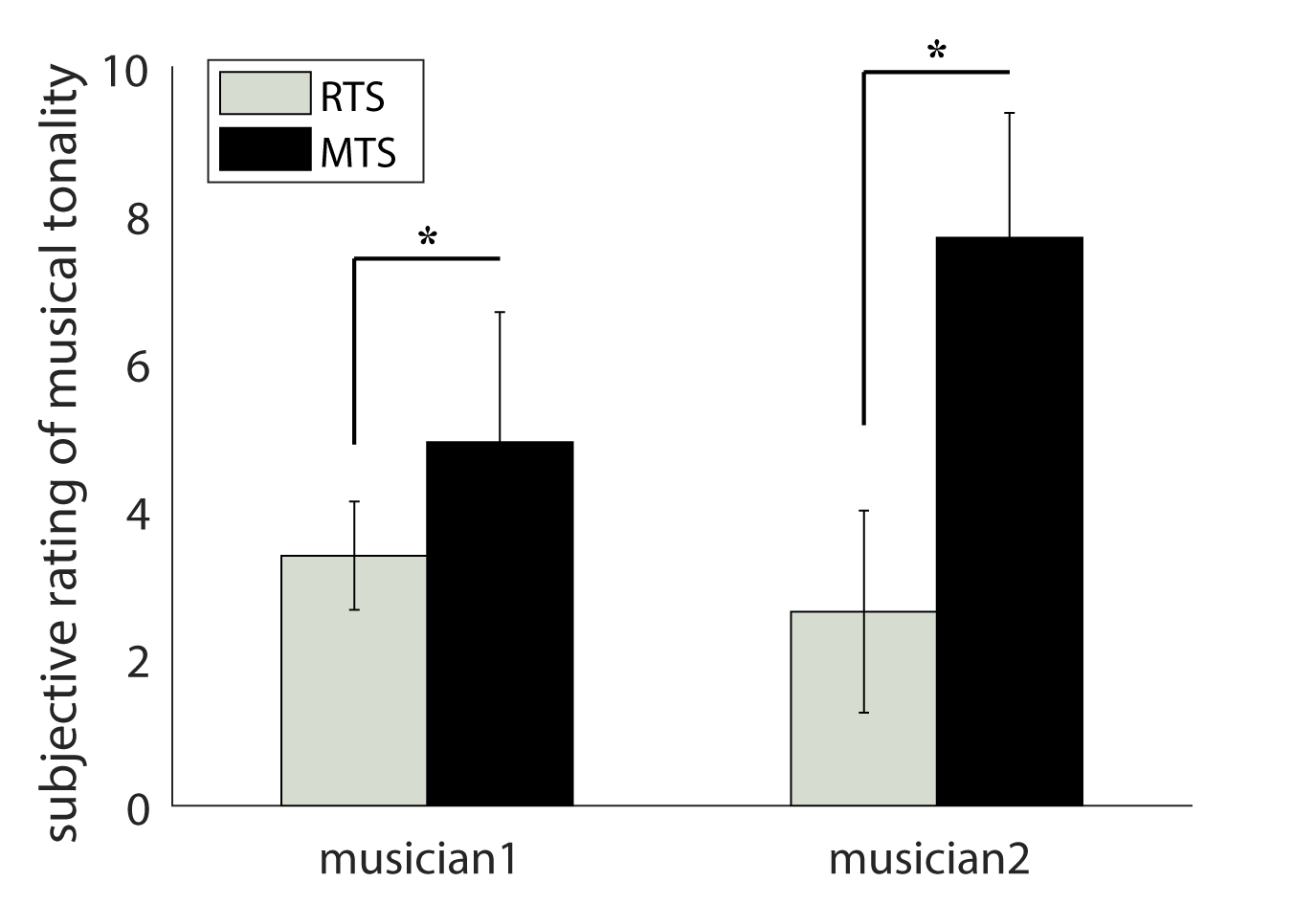

Supplement: S1 Fig — Y-axis: Average rating score. Scores are between 1–10, with 1 being least musical and 10 being most musical. Error bars are standard deviations. Dark bars: musical tonal sequences (MTSs). Gray bars: random tone sequences (RTSs). * p<0.001 (two sample t-test). (TIF) [file pone.0201765.s001.tif]

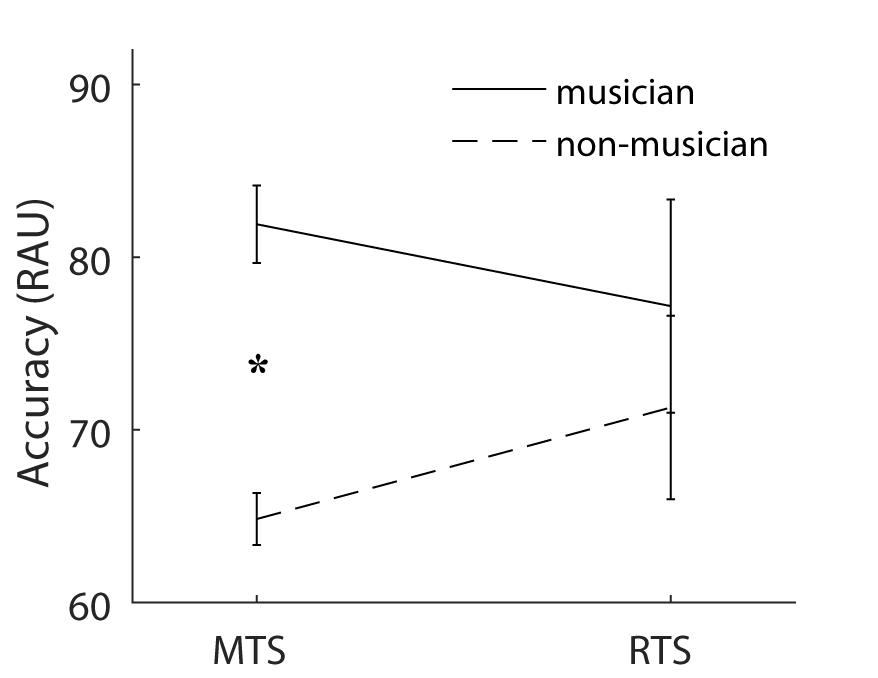

Supplement: S2 Fig — Musicians’ accuracy RAU value with MTSs was significantly higher (p<0.01) than that of non-musicians, whereas no significant difference was observed for test with RTSs. Error bars are standard errors. (TIF) [file pone.0201765.s002.tif]
